# Age-related changes of individual macular retinal layers among Asians

**DOI:** 10.1038/s41598-019-56996-6

**Published:** 2019-12-30

**Authors:** Jacqueline Chua, Yih Chung Tham, Bingyao Tan, Kavya Devarajan, Florian Schwarzhans, Alfred Gan, Damon Wong, Carol Y. Cheung, Shivani Majithia, Sahil Thakur, Georg Fischer, Clemens Vass, Ching-Yu Cheng, Leopold Schmetterer

**Affiliations:** 1 Singapore Eye Research Institute, Singapore National Eye Centre, Singapore, Singapore; 2 Academic Clinical Program, Duke-NUS Medical School, Singapore, Singapore; 3 SERI-NTU Advanced Ocular Engineering (STANCE), Singapore, Singapore; 4 Center for Medical Statistics Informatics and Intelligent Systems, Section for Medical Information Management and Imaging, Medical University Vienna, Vienna, Austria; 5 Department of Ophthalmology and Optometry, Medical University Vienna, Vienna, Austria; 6 Department of Ophthalmology, Yong Loo Lin School of Medicine, National University of Singapore and National University Health System, Singapore, Singapore; 7 Department of Ophthalmology, Lee Kong Chian School of Medicine, Nanyang Technological University, Singapore, Singapore; 8 Department of Ophthalmology and Visual Sciences, The Chinese University of Hong Kong, Shatin, Hong Kong; 9 Department of Clinical Pharmacology, Medical University Vienna, Vienna, Austria; 10 Center for Medical Physics and Biomedical Engineering, Medical University Vienna, Vienna, Austria

**Keywords:** Geriatrics, Three-dimensional imaging

## Abstract

We characterized the age-related changes of the intra-retinal layers measured with spectral-domain optical coherence tomography (SD-OCT; Cirrus high-definition OCT [Carl Zeiss Meditec]. The Singapore Epidemiology of Eye Diseases is a population-based, cross-sectional study of Chinese, Malays and Indians living in Singapore. Iowa Reference Algorithms (Iowa Institute for Biomedical Imaging) were used for intra-retinal layer segmentation and mean thickness of 10 intra-retinal layers rescaled with magnification correction using axial length value. Linear regression models were performed to investigate the association of retinal layers with risk factors. After excluding participants with history of diabetes or ocular diseases, high-quality macular SD-OCT images were available for 2,047 participants (44–89 years old). Most of the retinal layers decreased with age except for foveal retinal nerve fiber layer (RNFL) and the inner/outer segments of photoreceptors where they increased with age. Men generally had thicker retinal layers than women. Chinese have the thickest RNFL and retinal pigment epithelium amongst the ethnic groups. Axial length and refractive error remained correlated with retinal layers in spite of magnification correction. Our data show pronounced age-related changes in retinal morphology. Age, gender, ethnicity and axial length need be considered when establishing OCT imaging biomarkers for ocular or systemic disease.

## Introduction

During aging, the retina is susceptible to develop degenerative diseases, such as age-related macular degeneration^1,2^, glaucoma, and diabetic retinopathy^3^. Recent studies have highlighted the usefulness of mapping individual retinal layers in improving disease detection for age-related macular degeneration^4^, glaucoma^5,6^, diabetic macular edema^7^, and treatment monitoring of diabetic macular edema^8^. In light of the significance of individual retinal layer analysis, knowledge on their age-related differences in normal, healthy eyes is critical and useful in both the clinical and research settings.

Age-related changes in intra-retinal layers have been reported^9–16^, but have revealed somewhat contradictory results. For instance, one study showed thinning of RNFL^12^, whereas another reported thickening^9^ and yet another showed no correlation^13^ of RNFL with age. Most of these reports on the intra-retinal layer analysis were derived from clinic-based samples and thus may be less generalizable and subjected to selection bias. One recent study from Europe has performed intra-retinal layers analysis on a sample derived from the population and concluded that there was no age-related effect on RNFL^16^. However, this study only examined the age dependency using the mean thickness of each layer from the entire macular map. Thickness of individual retinal layers varies over the macular region, where inner retinal layers are thinnest and outer retinal layers are thickest in the foveolar subfield. Hence, majority of studies used the Early Treatment Diabetic Retinopathy Study (ETDRS) map^9–15^. Also, The Rhineland Study excluded eyes from patients with self-reported eye disease. Depending in part on self-reported eye disease may lead to the inclusion of eyes from patients with undiagnosed glaucoma and retinal/macular diseases. Last, the study is composed of subjects of European descent and may not be applicable to other ethnic/racial groups. Therefore, there has not yet been any analysis on the intra-retinal layers in a population-based study of normal, healthy eyes of another racial/ethnic group.

Based on spectral-domain optical coherence tomography (SD-OCT) findings, several investigators indicated thinner retina in eyes with longer eyeballs^17–19^. True retinal thickness may, however, be under-estimated in myopic eyes because of the ocular magnification effects related to OCT scanning^20^. We therefore have corrected for the magnification effects of SD-OCT on the thickness of individual retinal layers.

The purpose of the current study was to examine the age-related changes of the intra-retinal layers amongst three ethnic groups (Chinese, Malays and Indians), who are free of ocular diseases and diabetes in the population-based Singapore Epidemiology of Eye Diseases (SEED) Study, where the data were rescaled for the magnification effect of SD-OCT.

## Results

Of the 5,221 SEED participants (n = 9735 eyes) with OCT images, we excluded 3,772 eyes of participants with history of diabetes or presence of age-related macular degeneration^21^, glaucoma/-suspect/self-reported glaucoma^22^, or retinopathies^23^ or 2,565 eyes with poor quality OCT scans or 10 eyes with poor segmentations and 346 eyes due to missing clinical variables (Supplementary Fig. S1). This left 3,043 eyes of 2,047 participants for analysis, comprising of 1,431 eyes of 961 Chinese, 725 eyes of 485 Malays, and 887 eyes of 601 Indians. Older age, female gender, Indians/Malays (compared to Chinese), hyperlipidemia and hypertension are associated with higher odds of being excluded from the analysis (P < 0.001; Supplementary Table 1) There was no difference in terms of their ocular factors such as corneal curvature, axial length, refractive error and optic disc area (P > 0.05).

Table 1 shows the characteristics of the 2,047 study participants. The mean age of the participants was 56 ± 8 (44–89) years and 50% were women. There were 961 (47%) Chinese, 485 (24%) Malay, and 601 (29%) Indian participants. There were significant differences in the characteristics among the three ethnic groups. Chinese persons were younger, more myopic, had lower prevalence of hyperlipidemia/hypertension, flatter cornea, longer eyes, and smaller-looking optic disc area than Malays or Indians.

**Table 1:** Clinical characteristics among the three ethnic groups.

	All	Chinese	Malay	Indian	P value*
Number of participants	2047	961	485	601	
**Demographic & systemic factors**					
Age, years	56 ± 8	54 ± 7	59 ± 7	58 ± 8	**<0.001**
Gender, female	1016 (50%)	465 (48%)	250 (52%)	301 (50%)	**0.508**
Hyperlipidemia	886 (45%)	357 (38%)	231 (49%)	298 (52%)	**<0.001**
Hypertension	1030 (50%)	433 (45%)	282 (58%)	315 (52%)	**<0.001**
**Ocular factors**					
Corneal curvature, mm	7.7 ± 0.3	7.7 ± 0.3	7.6 ± 0.2	7.6 ± 0.3	**0.004**
Axial length, mm	23.8 ± 1.2	24.1 ± 1.3	23.6 ± 1.1	23.6 ± 1.0	**<0.001**
Refractive error, diopters	−0.4 ± 2.2	−0.9 ± 2.4	0.0 ± 2.0	+0.2 ± 1.9	**<0.001**
Optic disc area, mm^2^	1.9 ± 0.4	1.9 ± 0.4	2.0 ± 0.4	2.0 ± 0.4	**<0.001**

SD = standard deviation.

Data are number (%) or mean ± SD, as appropriate.

*P value was obtained with 1-way analysis of variance for continuous variables and with chi-square tests for categorical variables.

**Figure 1:**
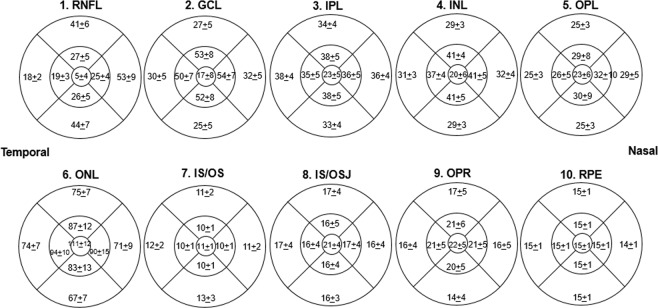
Diagram showing the thicknesses of the 10 individual retinal layers at different sectors. Data are mean ± standard deviation (μm).

The mean thickness values between the right and left eyes as well as their ICC are shown in Table 2. Comparing between right and left eyes across individuals, most of the intra-retinal layers were highly correlated (ICC > 0.75), others such as fovea RNFL, OPL at fovea and inner ring, and IS/OSJ at fovea and inner ring, fovea OPR were moderately correlated (ICC = 0.61–0.74) whereas only the fovea RPE was poorly correlated (ICC = 0.48). The relationships between the right and left eye scans of each participant were depicted using the Bland-Altman plots and the limits of agreement (Supplementary Fig. S2). Figure 1 shows the mean thickness of the individual retinal layers in the varying ETDRS subfields, where the RNFL at the fovea area was thinnest and the ONL at the fovea was thickest (5 μm vs 111 μm).

**Table 2:** Distribution of individual retinal layers thickness by right and left eyes (N = 992 participants).

	Right eye (mean ± SD)	Left eye (mean ± SD)	ICC between eyes (95% Conf. Interval)
**1. RNFL**			
Fovea	5.0 ± 4.2	5.7 ± 4.2	0.61 (0.56–0.66)
Inner ring	24.0 ± 3.6	24.6 ± 3.4	0.84 (0.81–0.87)
Outer ring	38.8 ± 5.5	39.1 ± 5.5	0.92 (0.91–0.93)
**2. GCL**			
Fovea	16.4 ± 7.9	17.1 ± 7.1	0.79 (0.77–0.82)
Inner ring	52.9 ± 6.4	52.6 ± 6.2	0.88 (0.86–0.89)
Outer ring	29.1 ± 3.9	28.8 ± 3.9	0.93 (0.92–0.94)
**3. IPL**			
Fovea	22.8 ± 4.8	23.3 ± 4.5	0.84 (0.82–0.86)
Inner ring	36.8 ± 3.9	37.2 ± 3.8	0.78 (0.75–0.81)
Outer ring	35.5 ± 3.0	35.5 ± 3.1	0.91 (0.90–0.92)
**4. INL**			
Fovea	19.4 ± 6.1	19.9 ± 5.1	0.75 (0.72–0.78)
Inner ring	40.0 ± 3.8	40.2 ± 3.7	0.90 (0.88–0.91)
Outer ring	30.3 ± 2.9	30.3 ± 2.9	0.96 (0.95–0.96)
**5. OPL**			
Fovea	23.2 ± 6.0	23.6 ± 6.3	0.66 (0.62–0.70)
Inner ring	29.3 ± 5.2	29.8 ± 5.1	0.72 (0.68–0.75)
Outer ring	26.0 ± 2.5	26.4 ± 2.6	0.84 (0.81–0.86)
**6. ONL**			
Fovea	110.6 ± 11.6	110.4 ± 11.4	0.84 (0.82–0.86)
Inner ring	88.8 ± 9.6	88.0 ± 9.7	0.90 (0.88–0.91)
Outer ring	71.9 ± 6.8	71.6 ± 6.9	0.96 (0.96–0.97)
**7. IS/OS**			
Fovea	11.1 ± 0.6	11.1 ± 0.6	0.82 (0.79–0.84)
Inner ring	10.2 ± 0.8	10.2 ± 0.7	0.89 (0.87–0.90)
Outer ring	11.7 ± 2.2	11.7 ± 2.2	0.96 (0.96–0.97)
**8. IS/OSJ**			
Fovea	21.3 ± 3.9	21.4 ± 4.0	0.66 (0.62–0.70)
Inner ring	16.2 ± 3.9	16.2 ± 4.0	0.68 (0.64–0.72)
Outer ring	16.5 ± 3.1	16.5 ± 3.0	0.79 (0.76–0.82)
**9. OPR**			
Fovea	22.1 ± 4.7	22.0 ± 4.6	0.74 (0.71–0.77)
Inner ring	21.3 ± 4.9	21.0 ± 4.9	0.77 (0.74–0.80)
Outer ring	16.2 ± 4.1	16.1 ± 4.0	0.86 (0.84–0.88)
**10. RPE**			
Fovea	14.6 ± 0.4	14.6 ± 0.4	0.48 (0.42–0.54)
Inner ring	14.7 ± 0.3	14.7 ± 0.3	0.80 (0.77–0.82)
Outer ring	14.6 ± 0.3	14.6 ± 0.3	0.93 (0.92–0.94)
**Macular thickness**			
Fovea	251.8 ± 24.8	254.5 ± 23.7	0.89 (0.87–0.90)
Inner ring	319.4 ± 17.1	319.9 ± 16.8	0.94 (0.94–0.95)
Outer ring	276.1 ± 14.5	276.0 ± 15.1	0.96 (0.95–0.96)

ICC = intraclass correlation.

Layers 1–10 (top to bottom; as defined by the software): 1. retinal nerve fiber layer (RNFL); 2. ganglion cell layer (GCL); 3. inner plexiform layer (IPL); 4. inner nuclear layer (INL); 5. outer plexiform layer (OPL); 6. outer nuclear layer (ONL); 7. photoreceptor inner/outer segments (IS/OS); 8. inner/outer segment junction to inner boundary of outer segment photoreceptor/retinal pigment epithelium complex (IS/OSJ to IB_RPE); 9. outer segment photoreceptor/retinal pigment epithelium complex (OPR); 10. retinal pigment epithelium (RPE).

*Total macular thickness is defined as layer 1 (retinal nerve fiber layer; RNFL) to layer 9 (outer segment photoreceptor/retinal pigment epithelium complex; OPR).

**Figure 2:**
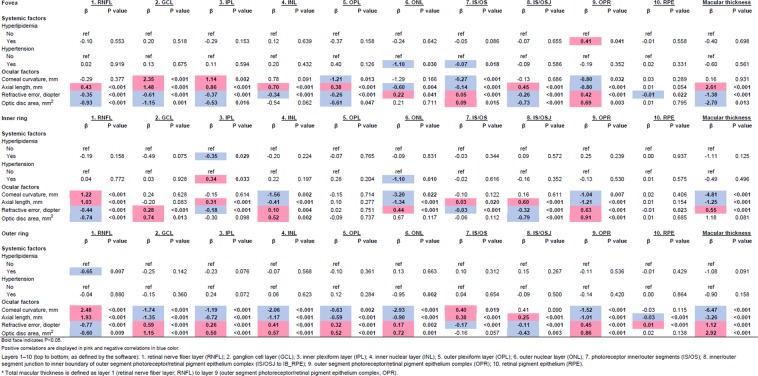
Age, gender and ethnicity adjusted associations of varying factors to individual retinal layers thickness (n = 3,043; N = 2,047).

**Figure 3:**
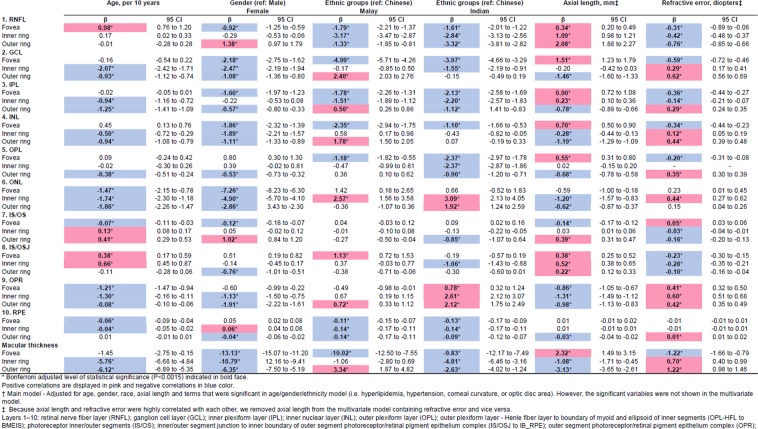
Multivariate associations of varying factors to individual retinal layers thickness (n = 3,043; N = 2,047).

**Figure 4:**
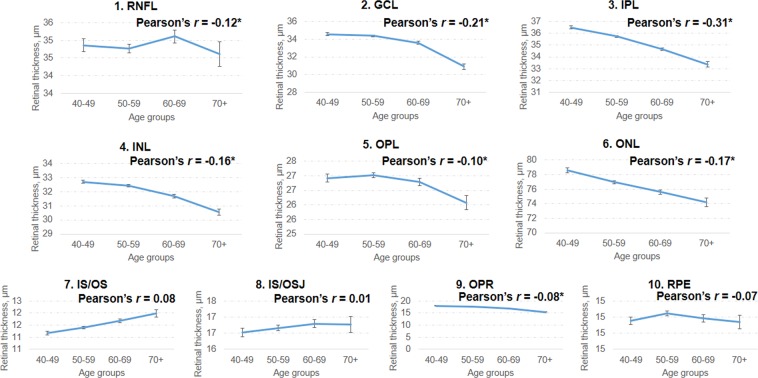
Line graphs showing the correlation of 10 individual retinal layers with age. Pearson’s correlation coefficients indicate the strengths of the linear relationship between the variables and asterisks (*) indicate Bonferroni corrected P value < 0.0015. Most retinal layers reduced with age except for layer 7 (IS/OS), 8 (IS/OSJ) and 10 (RPE). Data shown are after adjustment for age, gender, race, hyperlipidemia, hypertension, systolic blood pressure, diastolic blood pressure, intraocular pressure, corneal curvature, and axial length.

Figure 2 shows the association between various systemic/ocular factors with individual retinal layers after adjustments of age, gender and ethnicity. Variables found to be significant in the age- gender- ethnicity-model were adjusted in the multivariate regression modeling but not shown in the multivariate model (Fig. 3; Bonferroni corrections; P < 0.0015; Positive correlations are displayed in pink and negative correlations in blue color.). Majority of the macular layers decreased significantly with age and these were namely, i.e. GCL (β = −0.93 to −2.07 µm per decade; *r* = −0.09 to −0.24), IPL (β = −0.94 to −1.25 µm per decade; *r* = −0.11 to −0.30), INL (β = −0.50 to −0.94 µm per decade; *r* = −0.14), OPL (β = −0.38 µm per decade; *r* = −0.10), ONL (β = −1.47 to −1.86 µm per decade; *r* = −0.17), and RPE (β = −0.04 to −0.06 µm per decade; *r* = −0.18 to −0.20). However, some macular layers increased with age were RNFL at fovea (β = 0.98 µm per decade; *r* = 0.11), IS/OS at inner/outer rings (β = 0.13 to 0.41 µm per decade; *r* = 0.10) and IS/OSJ at fovea/inner rings (β = 0.38 to 0.66 µm per decade; *r* = 0.09). Figure 4 further showed the distribution of individual retinal layers among the varying age groups (entire ETDRS map) independent of age, gender, race, hyperlipidaemia, hypertension, systolic blood pressure, diastolic blood pressure, intraocular pressure, corneal curvature, and axial length. Most retinal layers showed a negative correlation with age, except for layer 7 (IS/OS), 8 (IS/OSJ) and 10 (RPE).

Men generally had thicker retinal layers than women except for these layers/sectors: RNFL, IS/OS at outer ring and RPE at inner ring. Chinese had the thickest RNFL (β = 1.33 to 3.32 µm thicker than Malays/Indians) and RPE (β = 0.09 to 0.14 µm thicker than Malays/Indians). Axial length and refractive error were associated with most of the retinal layers. In eyes with longer eyeball or greater myopia, most of the retinal layers thickened at the fovea and thinned at the inner/outer macula. At the fovea, tissues that thickened were namely the RNFL (β = 0.34 µm), GCL (β = 1.51 µm), IPL (β = 0.90 µm), INL (β = 0.70 µm), OPL (β = 0.55 µm), outer segment (β = 0.38 µm). At the inner/outer ring, tissues that thinned were namely the GCL (β = −1.46 µm), IPL (β = −0.78 µm), INL (β = −0.28 to −1.19 µm), OPL (β = −0.68 µm), ONL (β = −0.62 to −1.20 µm) and the OPR (β = −0.98 to −1.31 µm). The corresponding similar correlation can also be seen for the refractive error component. Patients who received cataract surgery will often receive an intraocular lens implant (n = 38 eyes of 29 persons), hence their refractive error status may not necessarily represent their ocular biometry. For example, a highly myopic individual who underwent cataract surgery may now be emmetropic. However, he/she will still have a long eyeball and there might be residual confounding effect by history of cataract surgery which may impact the comparison of retinal layers between refractive error status. To minimize this potential residual confounding effect, we further performed a sensitivity analysis limited to subjects without any history of cataract surgery and still observed significant correlations with refractive error status.

## Discussion

Individual retinal layers are routinely generated from OCT images^24^ and can be automatically segmented and measured with the Iowa Reference Algorithms^25–27^. In this population-based study, we applied a publicly available OCT software to segment the ten retinal layers and examined the effect of age on specific retinal layers in normal and healthy participants. We showed that the thickness of individual retinal layers varies by age. Most of the retinal layers decreased with age except for foveal RNFL and the inner and outer segments of photoreceptors, where they increased with age. We also found the influence of gender, ethnicity and axial length on specific retinal layers to be sectoral-specific.

### Age

Aging has been shown to be associated with decreased areas of dendritic and axonal arbors and decreased density of cells and synapses in the retina^28,29^. Our findings are consistent with reports of thinning of these retinal layers, namely GCL^9,12,14,16^, IPL^9,12,14,16^, INL^12,16^, OPL^9^, ONL^9,16^, and RPE^9,30^, in the literature. However, direct comparisons among these studies remain challenging^9–16^. Apart from the issues mentioned in the introduction section, most of these studies were clinic-based with small sample sizes (N < 525)^9–15^, different study design i.e. absence of ophthalmological examination^16^, or the use of different SD-OCT machines i.e. Topcon 3D OCT (OCT-1000; Topcon, Tokyo, Japan)^12,14^, or Heidelberg Spectralis OCT (Heidelberg Engineering, Heidelberg, Germany)^9,10,13,15,16^ and different segmentation algorithms i.e., custom software^12^, Iowa Reference Algorithms (Iowa Institute for Biomedical Imaging)^14^, and Heidelberg Eye Explorer (HEYEX)^9,10,13,15,16^ or the reporting retinal thickness for the entire macular scan^16^ instead of various ETDRS subfields. Last, the inadequate considerations for statistical analysis i.e. absence of Bonferroni correction during multiple comparisons of retinal thickness at various ETDRS subfields^9–12,15^.

We found that the foveal RNFL and the inner and outer segments of photoreceptors increased with age. The average RNFL thickness at the inner ring of superior, inferior, nasal, and temporal to the foveola was 27, 26, 25, and 19 µm, respectively. The topographical variation of RNFL reported in this study is similar to a histological study of normal human eyes where that average RNFL thickness that is superior, inferior, nasal, and temporal to the foveola was 27, 34, 26, and 12 µm, respectively^31^. The finding of a thickened foveal RNFL with age (β = 0.98 µm per decade) as seen by us has also been reported in Chinese eyes (β = 1.3 µm per decade)^9^. Mauschitz *et al*. also reported a positive correlation between foveal RNFL with age in Caucasian eyes (β = 0.84 µm per decade), albeit not reaching statistically significance. This effect is unlikely to be associated with an increase in nerve fibers, because circumpapillary RNFL is known to decline with age^32,33^. Rather, we speculate that the thickening of RNFL with age is a reflection of Müller glial cells activity. The RNFL contains the axonal nerve fibers of retinal ganglion cells and the radial processes of Müller glial cells forming endfeet at the inner limiting membrane. Immunocytochemical study on normal retina have shown a general increase of glial fibrillary acid protein (GFAP) immunoreactive Müller glial cells with age^34^. The OCT finding of a thickened foveal RNFL with age may represent the accumulation of GFAP of an ageing retina. Hence, the age-related RNFL decline may have masked by the thickening of Müller glial cells. Even though age has a significant effect on foveal RNFL, one needs to interpret the foveal RNFL thickness data with caution. This is because the thickness of RNFL in foveal area is very thin, about 5 µm, which is close to the axial resolution of the Cirrus OCT instrument^35^. Also, the effect of 1 µm for every 10 years is small and does not appear to be relevant.

Fewer studies have examined the effects of aging on the inner and outer segments of photoreceptors^12,14^. We saw a positive correlation of inner and outer segments of photoreceptors with age, which is in agreement with Ooto *et al*.^12^. The outer segment layer may thickened because of the decreased ability of the retinal pigment epithelium to phagocytose with age^36^. Demirkaya instead reported a thinning of outer segments with age^14^. Such differences may be also due to the very thin layer of inner and outer segments of photoreceptors which is difficult to detect accurately.

### Gender

The expected gender-related differences in individual retinal layers were found in the present study. We found that men generally had thicker retinal layers than women. This is in agreement with previous studies which also reported a gender-related differences in the thicknesses of individual retinal layers in Japanese^12^ and Caucasians^13,16^. These studies showed men had a thicker inner nuclear layer and outer nuclear layer than women^12,13^. Another study also showed a gender difference at the ganglion cell layer^16^, which is also consistent with our findings. Apart from the neuronal cell bodies, we further showed that both the inner and outer plexiform layers showed gender differences. This implies that the gender differences in retinal architecture extend to both the cell bodies and dendrites which explains the thicker mean retinal thickness in men than women^17,18,37^. Several regions of the brain have been shown to contain a higher neuronal densities and neuronal number estimates^38^ as well as in higher synaptic density^39^ in males compared with females. The gender differences in the retinal nuclear and plexiform layer thicknesses could possibly be explained by the anatomical similarities between the retina and brain. Gender differences in the individual retinal layers are of interest because it further supports the notion of accounting for gender in the OCT normative database when conducting analyses of OCT measurements and might help to explain the significant number of women in the macular hole population may be related to their relatively thin retinas^40,41^.

### Race/ethnic groups

Our study found ethnic differences of individual retinal layers between Singaporean Chinese, Malays and Indians. In particular, the differences in retinal layer thickness between Indians and Chinese were found to be statistically significant for many of the macular layers, and potentially clinically important due to its high standardized β values. Given that Asians are a heterogeneous population, it is not unexpected that variations in individual retinal layers exist within ethnic subgroups among Asians. Ethnic differences on macular thickness is relatively well characterized and are incorporated into the normative databases of commercially available OCT devices^17,42^. Controlling for ethnic subgroup differences might improve the diagnostic capability of the OCT when using it for individual retinal layer analysis.

### Axial length/refractive error

Axial length has been shown to influence the measurements of individual macular layer thickness^12,13,43,44^. However, none of these studies have accounted for the ocular magnification effects of OCT, which has a profound effect on the “true” retinal thickness^20^. Only one study corrected for the magnification effects of OCT and reported that the relationship between axial length and inner retinal tissues was abolished whereas the negative correlation between axial length outer retinal tissues remained. They suggested that only the outer retina was affected by axial elongation whereas the inner retina remained unaffected by axial elongation^45^. However, our results are in contrast to Higashide and co-workers^45^. Instead, we show that the relationship between axial length/ refractive error remains significant for most of the macular layers even after magnification correction, which implies that axial elongation affects both the inner and outer retinal layers indiscriminately. Several differences in study design could have contributed to these inconsistent results, such as sample sizes and ethnicity of study participants. The larger sample size in our study (N = 2,000 vs 200), which will lend statistical power to detecting weaker associations of the thinner inner retinal layers. Another reason might be the ethnicity of sample (Chinese, Malays and Indians vs Japanese). Asians are a heterogeneous population, and variations in retinal layers may exist within ethnic subgroups. In addition, we now show that most of the macular layers correlate with axial length/refractive error similarly. In eyes with longer eyeball or greater myopia, most of the retinal layers thickens at the fovea and thins at the inner/outer macula. This is also consistent with previous findings, where eyes with longer axial length has a thicker central fovea, but thinner inner and outer macula^17–19^. This is in support of the theory proposed by Wu and co-worker^46^, where the axial elongation of the eye, results in peripheral retinal thinning and the centripetal force of the posterior vitreous leads to the elevation of the fovea. Another reason why an association remains with axial length/refractive error may be that the Littmann’s formula to correct for ocular magnification remains insufficient. Although this method was proven to be similarly accurate to more detailed calculations using additional ocular biometric parameters^47^, under- or overcorrections may occur in eyes where ocular dimensions deviate from the assumption in the formula. We had previously showed that ocular biometry such as corneal curvature also had an impact on the RNFL thickness^32^.

The strength of our study is that it gives a better understanding of age-related variation in individual retinal sublayers by comparing the thickness of individual retinal layers of various ages and axial lengths obtained from a large population-based sample, in which participants received ophthalmological examination and imaged with the same OCT machine in the same clinical setting. A potential weakness is that the study is a cross-sectional design, and thus does not provide the age-related thinning rate of retinal layers. Second, because participants excluded from the analysis were older, women, of Indian ethnicity, our results may not be fully generalizable to the general population. However, there was no differences in the ocular factors between those who were excluded and included from the analysis. Third, all participants were of the three major Asian ethnic groups, and thus may not be directly applied to other racial/ ethnic groups. Last, the segmentation of each layer in all B-scan images was neither manually nor automatically checked. However, the Iowa Reference Algorithm produces retinal thickness metrics that is comparable to the manual measurements of OCT images by retinal specialists^48^. We also excluded OCT scans with a signal strength less than 6 and/or significant movement artifacts, excluded scans with outliers and excluded participants with eye diseases, relying on a combination of self-reported and of medical diagnoses. Approximately <5% of the entire sample had signal strength of 6. We reran the analysis by excluding OCT scans with signal strength of 6 and the findings remained similar.

In summary, the data presented in this study show a substantial difference in the structure of individual retinal layers with advancing age. Furthermore, our results suggest that these differences are impacted by gender, ethnicity and axial length. Given that the subjects in the present study were free from eye diseases and diabetes, the results represent true age-related changes. Further studies are required to better understand that the mechanisms underlying the age-, gender- and ethnicity-dependence of retinal morphology.

## Methods

### Study participants

The data for this study were derived from the Singapore Epidemiology of Eye Disease (SEED) study, comprising of Chinese, Malay and Indians aged 40 to 80 years. Details of the study methodology were identical and have been reported elsewhere^49–51^. Data were derived from 2,047 participants, of which 961 were Chinese (year 2009–2011), 485 Malays (year 2010–2014) and 601 Indians (year 2013–2015). Ethics approval was obtained from the SingHealth Centralized Institutional Review Board. Written, informed consent was obtained for all participants in adherence to the Declaration of Helsinki.

### Ocular examinations

Participants underwent an ocular examination including visual acuity, subjective refraction, slit-lamp biomicroscopy, gonioscopy, intraocular pressure (IOP) measurement using Goldmann applanation tonometry, measurement of central corneal thickness using an ultrasound pachymeter (CCT Advent; Mentor O & O Inc., Norwell, USA), corneal curvature and refractive error using an autorefractor (Canon RK-5 Autorefractor Keratometer; Canon Inc., Japan), axial length using non-contact partial coherence interferometry (IOL Master V3.01, Carl Zeiss Meditec AG, Germany) and posterior segment examination at the slit-lamp using a 78 Diopter lens^52^.

### Other measurements

Detailed interviewer-administered questionnaire was used to collect demographic data, medication and ocular surgery histories. Blood pressure was measured using a digital automatic blood pressure monitor (Dinamap model Pro Series DP110X-RW, GE Medical Systems Information Technologies, Inc., Milwaukee)^53^. Non-fasting venous blood samples were collected for biochemistry analysis. Diabetes was defined as random glucose of ≥ 11.1 mmol/l, diabetic medication usage, physician diagnosis of diabetes, or serum HbA1c ≥ 6.5%. Hypertension was defined as systolic blood pressures ≥ 140 mmHg or diastolic blood pressures ≥ 90 mmHg or physician diagnosed hypertension or self-reported history of hypertension. Hyperlipidemia was defined as total cholesterol ≥ 6.2 mmol/L or self-reported use of lipid lowering drugs.

### Optical coherence tomography imaging

Participants underwent Cirrus SD-OCT (Carl Zeiss Meditec, Inc, Dublin, CA) imaging after pupil dilation to acquire a 200 × 200 macular and optic disc cube scans in each eye^17^. Trained graders masked to the participant characteristics reviewed the quality of OCT scans. Poor quality images (signal strength less than 6 and/or significant movement artifacts) were excluded from the analysis.

### Automated analysis of retinal thickness

**Figure 5:**
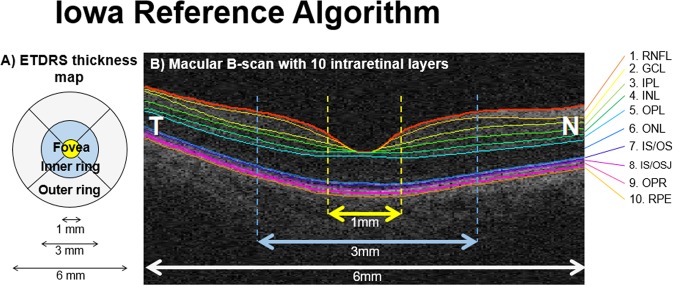
ETDRS grid and macular B-scan with 10 individual retinal layers of the right eye. Standard ETDRS grid showing the foveal subfield (yellow). The inner ring is an average of the four parafoveal subfields (blue) and the outer ring of the four perifoveal subfields (white). (**B**) A screenshot of 10 layer (11 boundary) segmentation of an OCT image, produced by the Iowa Reference Algorithms as indicated by the colored lines and corresponding retinal layers. Layers 1–10 (top to bottom; as defined by the software): 1. retinal nerve fiber layer (RNFL); 2. ganglion cell layer (GCL); 3. inner plexiform layer (IPL); 4. inner nuclear layer (INL); 5. outer plexiform layer (OPL); 6. outer nuclear layer (ONL); 7. photoreceptor inner/outer segments (IS/OS); 8. inner/outer segment junction to inner boundary of outer segment photoreceptor/retinal pigment epithelium complex (IS/OSJ to IB_RPE); 9. outer segment photoreceptor/retinal pigment epithelium complex (OPR); 10. retinal pigment epithelium (RPE).

Macular OCT images were directly imported into the automatic OCT layer segmentation algorithm (Retinal Image Analysis Lab, Iowa Institute for Biomedical Imaging, Iowa City, IA)^25–27^. The accuracy^48^ and reproducibility^54,55^ of the Iowa Reference Algorithms analysis software have previously been reported in patients with diabetic macular oedema and in healthy volunteers of varying ages. Mean retinal thickness values of 10 retinal layers were obtained on all images for the foveal subfield and the inner and outer rings of a standard ETDRS grid (Fig. 5). Total retinal thickness was also calculated, as the distance from the most anterior hyper-reflective line (corresponding to the inner limiting membrane; ILM) to the posterior of the outermost hyper-reflective line (corresponding to the inner boundary of RPE). Measurements of optic disc area were extracted from the optic disc scans.

Lastly, we cleaned the IOWA generated data by removing the outliers above or below standard deviations (SD) when compared to the Cirrus generated OCT full retinal thickness measurement (n = 10 eyes removed). Supplementary Fig. S3 show the scatter plots (A–C) and Bland-Altman plots (D–F) of full retinal thickness measurements generated by Cirrus and Iowa Reference Algorithms. There was an excellent agreement between the full retinal thickness measurement from Cirrus and IOWA (all *r* > 0.94 and ICC > 0.93). The full retinal thickness generated by IOWA was 2.7 µm and 1.5 µm thicker than Cirrus in the fovea, and outer rings, respectively (P < 0.001) whereas there was no difference in the inner ring (P = 0.088).

### Adjustment for ocular magnification

We further corrected the ocular magnification effect associated with OCT scans by rescaling the sizes of three concentric circles^45,56^. Briefly, the Littmann’s formula was used, expressed as *s*_corrected_ = *p* × *q* × *s*^57^, where *s*_corrected_ is the actual fundus dimension, *s* is the scanning size of the protocol obtained using OCT, *p* is the magnification factor for the camera of the imaging system, and *q* is the magnification factor for the eye. According to the calculation scheme of Bennett, the ocular magnification factor *q* of the eye can be determined with the formula *q* = 0.01306 × (axial length − 1.82)^58^. Further, *p* is a constant in a telecentric system, and the *p* of the Cirrus system is 3.382^59^. Therefore, the actual size (*s*) of the 1-, 3- and 6-mm scan area in the macular cube scan can be calculated using this formula. According to the formula, the magnification corrected scanning size is calculated as:

$${s}_{{\rm{corrected}}}=3.382\times 0.01306\times ({\rm{axial}}\,{\rm{length}}-1.82)\times s$$

A magnification-corrected analytical area was determined for each scan when we entered the data of axial length into the formula. For example, the 20 × 20° square scan area is supposed to be a nominal 6×6 mm square area for all eyes when magnification correction is not considered. The scan area corresponded to a 7×7 mm square area after magnification correction in an eye with an axial length of 28.25 mm. The sizes of three concentric circles were then rescaled according to the dimension of the ‘magnification-corrected’ scan area^45,56^.

### Statistical analyses

Primary outcomes were thickness measurements of intra-retinal layers. For individual-level analysis, 1-way analysis of variance (ANOVA) was performed to compare among ethnic groups for continuous variables, and chi-square tests were used for categorical variables. For eye-level comparisons between ethnic groups, we used a linear mixed model with a random intercept term to account for the effect of individual (between-eye correlation) with a post-hoc likelihood ratio test to compare nested models with and without ethnicity. Model parameters were estimated using maximum likelihood. We also evaluated the correlation of the intra-retinal thickness measurements between eyes, using intraclass correlation coefficients (ICC) and values less than 0.5, between 0.5 and 0.75, between 0.75 and 0.90, and greater than 0.90 indicate poor, moderate, good, and excellent correlation, respectively^60^. We also measured the strength of the association between the various intra-retinal thickness measurements with age using the Pearson’s *r* correlation coefficient. We explored the differences between the right and left eye scans of each participant using the using the Bland-Altman plots and presented the limits of agreements.

Associations between ocular and systemic factors with intra-retinal layers were assessed using age-gender-ethnicity-model and multivariate linear regression models with generalized estimating equations to account for the correlation between pairs of eyes for each individual. Covariates such as hyperlipidemia, hypertension, corneal curvature, axial length, refractive error, optic disc area were considered because they were related to macular thickness^17,19,37,61^. These covariates were adjusted in the multivariate model if they had a statistical significance of P < 0.05 in the first model. Because axial length and refractive error were highly correlated with each other and had high variance inflation factors (VIF > 5), we removed axial length from the multivariate model containing refractive error. To avoid α error accumulation due to multiple testing, we used a conservative Bonferroni correction and considered results statistically significant at the level α = 0.05/33 = 0.0015. Data were analyzed with statistical software (STATA, version 13.1; StataCorp LP).

## Supplementary information

Supplementary information.

## Data Availability

The datasets generated during and/or analyzed during the current study are not publicly available due to the terms of consent to which the participants agreed but are available from the corresponding author on reasonable request.
