# Supplementary material for: Age-related changes of individual macular retinal layers among Asians
Source: Sci Rep. 2019 Dec 30;9:20352. doi: 10.1038/s41598-019-56996-6 (PMC6937292; doi:10.1038/s41598-019-56996-6)
Supplement: Supplementary file 1 — Supplementary information. [file 41598_2019_56996_MOESM1_ESM.pdf]

Age-related changes of individual macular retinal layers among Asians

Jacqueline Chua, BOptom, PhD,[1,2] Yih Chung Tham, PhD,[1,2] Bingyao Tan, PhD,[1,3] Kavya Devarajan, M. Eng,[1,3] Florian Schwarzhans, MSc,[4] Alfred Gan, BSc,[1] Damon Wong, PhD,[3,8] Carol Y. Cheung, PhD,[7] Shivani Majithia, OD,[1] Sahil Thakur, MS,[1] Georg Fischer, MSc,[4] Clemens Vass, MD,[5] Ching-Yu Cheng, MD, PhD,[1,2,6] Leopold Schmetterer, PhD,[1-3, 8-10]

Supplementary Figure S1. Flow chart of the inclusion and exclusion criteria of study eyes

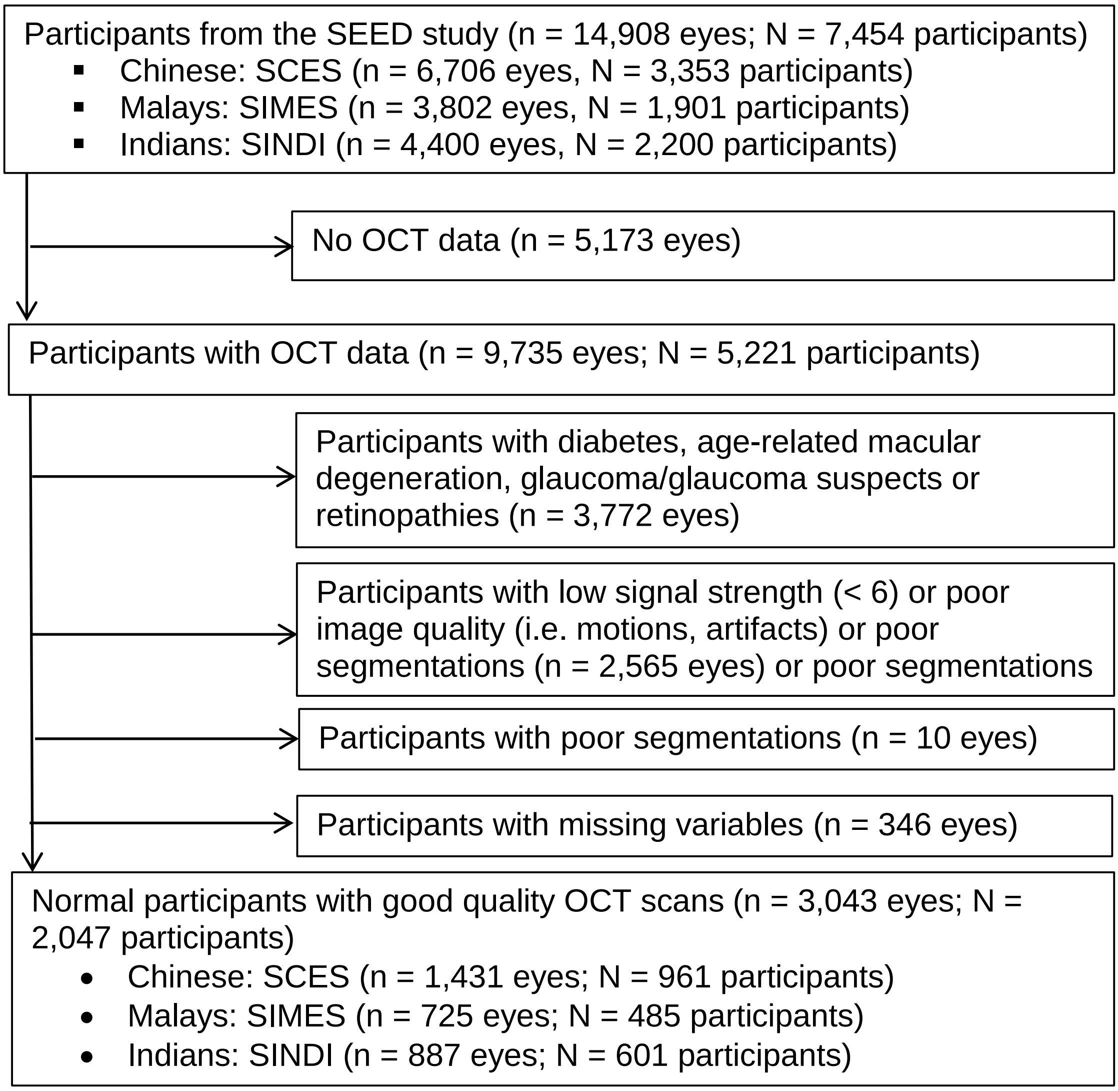

Supplementary Figure S2. Bland-Altman plots of individual retinal thickness measurements between the right and left eye.

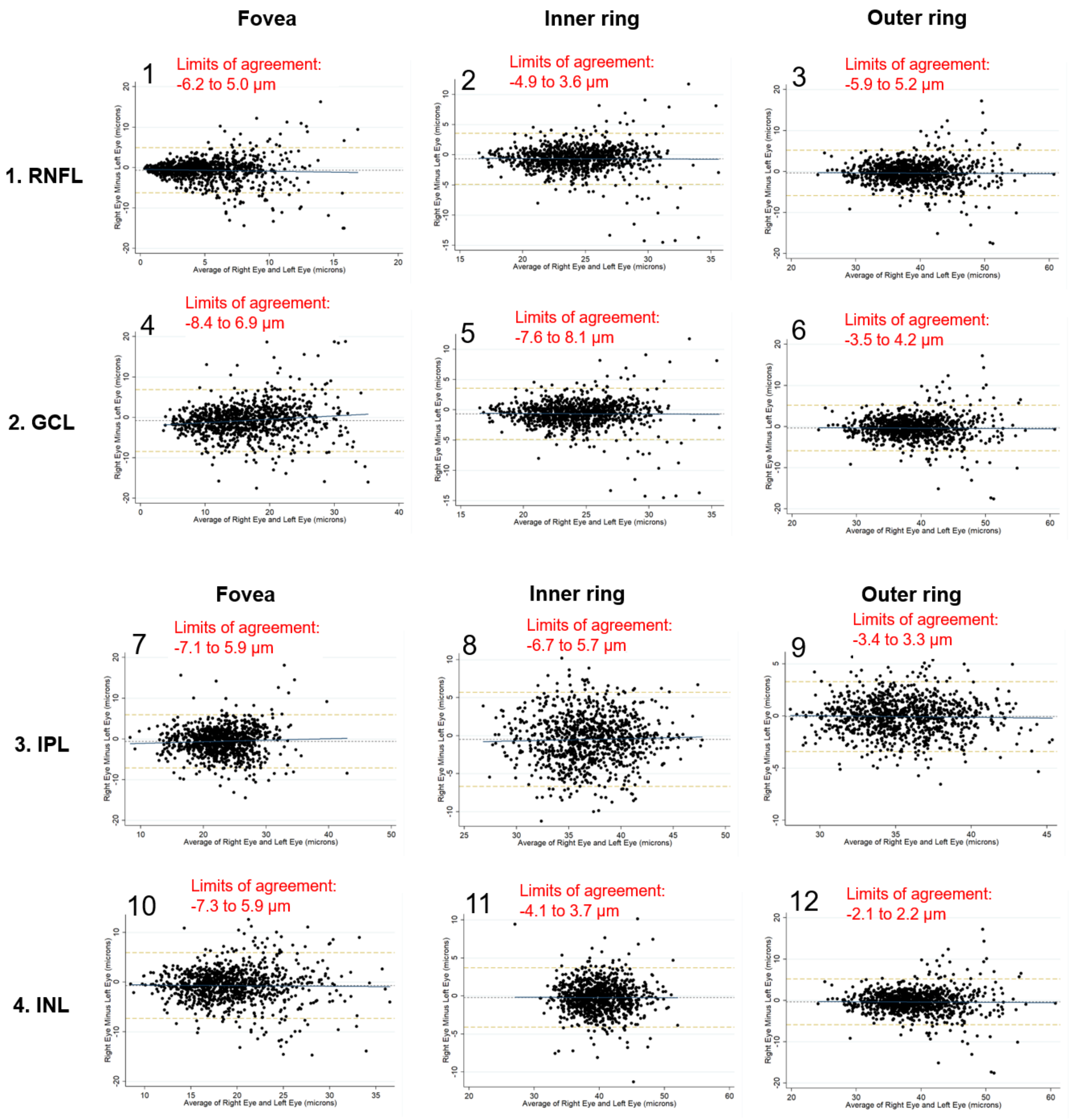

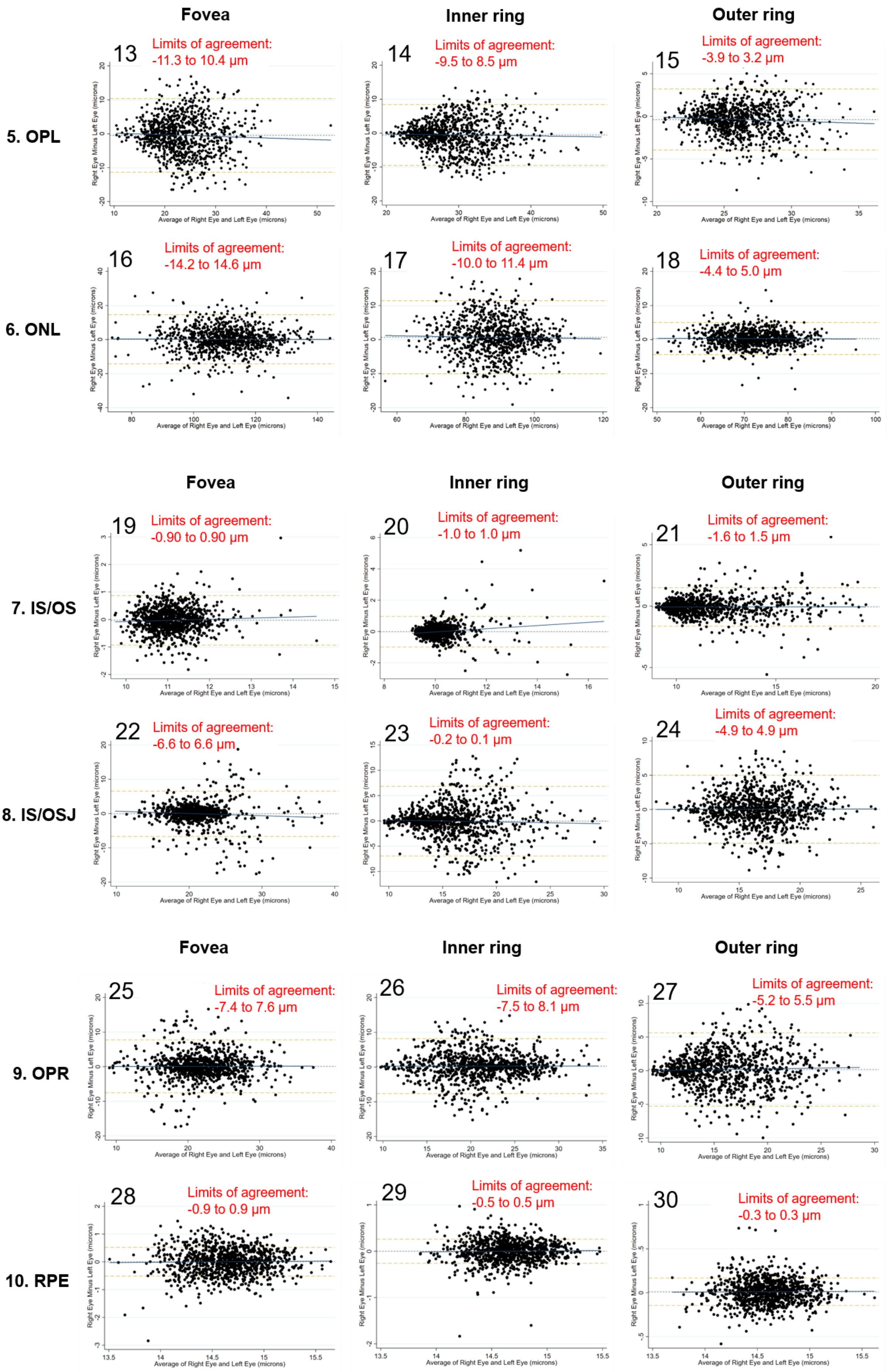
Fovea
Inner ring
Outer ring
5. OPL
13
Limits of agreement: -11.3 to 10.4 µm
14
Limits of agreement: -9.5 to 8.5 µm
15
Limits of agreement: -3.9 to 3.2 µm
6. ONL
16
Limits of agreement: -14.2 to 14.6 µm
17
Limits of agreement: -10.0 to 11.4 µm
18
Limits of agreement: -4.4 to 5.0 µm
Fovea
Inner ring
Outer ring
7. IS/OS
19
Limits of agreement: -0.90 to 0.90 µm
20
Limits of agreement: -1.0 to 1.0 µm
21
Limits of agreement: -1.6 to 1.5 µm
8. IS/OSJ
22
Limits of agreement: -6.6 to 6.6 µm
23
Limits of agreement: -0.2 to 0.1 µm
24
Limits of agreement: -4.9 to 4.9 µm
Fovea
Inner ring
Outer ring
9. OPR
25
Limits of agreement: -7.4 to 7.6 µm
26
Limits of agreement: -7.5 to 8.1 µm
27
Limits of agreement: -5.2 to 5.5 µm
10. RPE
28
Limits of agreement: -0.9 to 0.9 µm
29
Limits of agreement: -0.5 to 0.5 µm
30
Limits of agreement: -0.3 to 0.3 µm
Right Eye Minus Left Eye (microns)
Average of Right Eye and Left Eye (microns)

Supplementary Figure S3. Scatter plots (A-C) and Bland-Altman plots (D-F) of full retinal thickness measurements generated by Cirrus and Iowa Reference Algorithms.

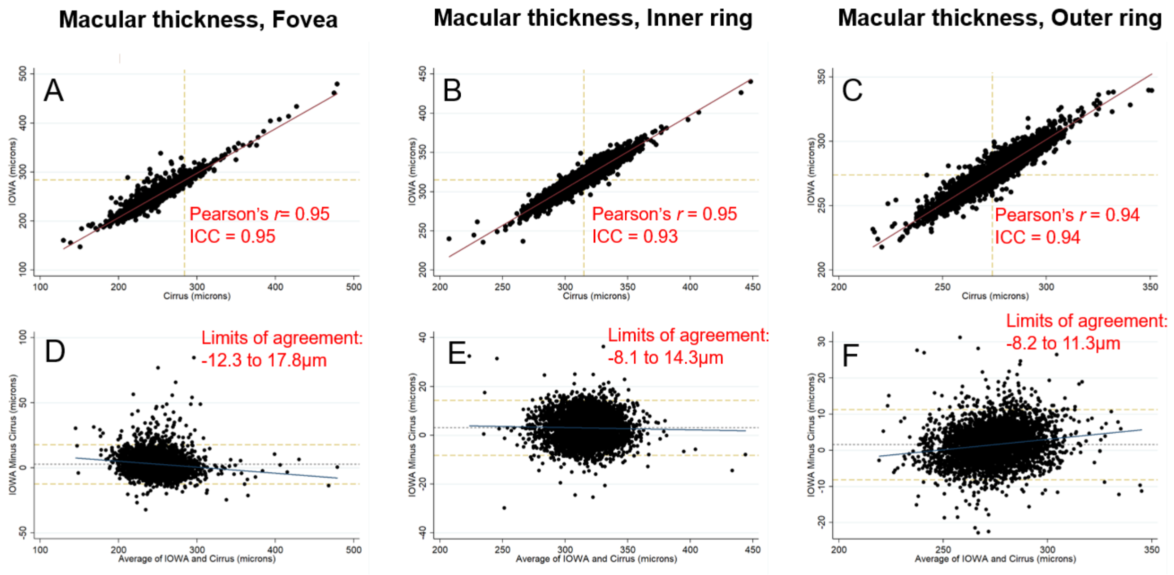

Supplementary Table 1: Associations of participants excluded from the analysis with demographics, medical and ocular characteristics.

Characteristics	Odds Ratio (95% confidence interval) Multivariable adjusted	*P* value
Age, years	1.05 (1.04-1.06)	**<0.001**
Gender, male		
Male	Reference	
Female	1.28 (1.12-1.46)	**<0.001**
Ethnic groups		
Chinese	Reference	
Malay	1.79 (1.50-2.14)	**<0.001**
Indian	2.12 (1.80-2.50)	**<0.001**
Hyperlipidemia		
No	Reference	
Yes	1.40 (1.21-1.62)	**<0.001**
Hypertension		
No	Reference	
Yes	1.44 (1.25-1.65)	**<0.001**
Corneal curvature, mm	0.86 (0.65-1.15)	0.310
Axial length, mm	1.02 (0.94-1.12)	0.573
Spherical equivalent refractive error, diopters	0.96 (0.92-1.00)	0.054
Optic disc area	0.89 (0.77-1.02)	0.104
